# Multiple Sites in αB-Crystallin Modulate Its Interactions with Desmin Filaments Assembled *In Vitro*

**DOI:** 10.1371/journal.pone.0025859

**Published:** 2011-11-09

**Authors:** Scott A. Houck, Andrew Landsbury, John I. Clark, Roy A. Quinlan

**Affiliations:** 1 Department of Biological Structure, University of Washington, Seattle, Washington, United States of America; 2 School of Biological and Biomedical Sciences, University of Durham, Durham, United Kingdom; 3 Department of Ophthalmology, University of Washington, Seattle, Washington, United States of America; 4 Biophysical Sciences Institute, University of Durham, Durham, United Kingdom; Dalhousie University, Canada

## Abstract

The β3- and β8-strands and C-terminal residues 155–165 of αB-crystallin were identified by pin arrays as interaction sites for various client proteins including the intermediate filament protein desmin. Here we present data using 5 well-characterised αB-crystallin protein constructs with substituted β3- and β8-strands and with the C-terminal residues 155–165 deleted to demonstrate the importance of these sequences to the interaction of αB-crystallin with desmin filaments. We used electron microscopy of negatively stained samples to visualize increased interactions followed by sedimentation assays to quantify our observations. A low-speed sedimentation assay measured the ability of αB-crystallin to prevent the self-association of desmin filaments. A high-speed sedimentation assay measured αB-crystallin cosedimentation with desmin filaments. Swapping the β8-strand of αB-crystallin or deleting residues 155–165 increased the cosedimentation of αB-crystallin with desmin filaments, but this coincided with increased filament-filament interactions. In contrast, substitution of the β3-strand with the equivalent αA-crystallin sequences improved the ability of αB-crystallin to prevent desmin filament-filament interactions with no significant change in its cosedimentation properties. These data suggest that all three sequences (β3-strand, β8-strand and C-terminal residues 155–165) contribute to the interaction of αB-crystallin with desmin filaments. The data also suggest that the cosedimentation of αB-crystallin with desmin filaments does not necessarily correlate with preventing desmin filament-filament interactions. This important observation is relevant not only to the formation of the protein aggregates that contain both desmin and αB-crystallin and typify desmin related myopathies, but also to the interaction of αB-crystallin with other filamentous protein polymers.

## Introduction

Human αB-crystallin is a small heat shock protein (sHSP) that interacts with a variety of important cellular proteins with the capacity to polymerise into either filaments [1], or tubules [2] or fibrils [3]. The fact that αB-crystallin is often part of the histopathological signature used to characterise a variety of human diseases [4] highlights the potentially important role that αB-crystallin plays in their etiology. This became apparent when it was discovered that mutations in both αB-crystallin (R120G; [5]) and desmin [6] can cause cardiomyopathy, typified by aggregates containing both proteins [4]. The R120G mutation in αB-crystallin also induced the aggregation of desmin filaments in transfected cells [7]. The dissociation constant was increased two fold for the R120G mutant compared to the wild-type αB-crystallin [7], which appeared to encourage the increased interaction of desmin filaments leading to their aggregation in transfected cells and in the muscles of affected individuals. Previously it had been established that αB-crystallin modulated the assembly of intermediate filaments [8] and reduced the extent of filament-filament interactions *in vitro* [9]. Over-expression of wild-type αB-crystallin is capable of reversing intermediate filament aggregation in transfected cells suggesting that αB-crystallin was involved in regulating the local associations of intermediate filaments [10]. The fact that mutations inαB-crystallin caused the aggregation of desmin filaments [5], [7] also supports this view. It is therefore important to identify the sequences in αB-crystallin that are responsible for the effects on intermediate filaments and particularly desmin because mutations in either can be the genetic basis of myopathy [11], [12], [13].

Pin array studies identified sequences in αB-crystallin involved in the recognition of a variety of different client proteins including desmin and GFAP, two examples of intermediate filament proteins [1]. These sequences were not unique to the interaction of αB-crystallin with either desmin or GFAP [1], evidence of the ability of αB-crystallin to recognise a wide range of potential protein clients [14], [15], [16], [17], [18], [19], [20], [21], [22], [23]. The five sequences in αB-crystallin with the strongest binding to desmin were spread throughout the primary sequence from the N- to the C-terminus and included some that were involved in αB-crystallin oligomerisation. The pin-array assays did not consider the assembly status of the desmin or GFAP, a potentially important factor in the mechanism of αB-crystallin activity. Indeed, the interaction between the αB-crystallin peptides and desmin was inversely correlated with temperature in the pin array studies [1]. In contrast, the fraction of αB-crystallin that pelleted with desmin filaments in the sedimentation assays increased with temperature [7]. Therefore it is important to verify that the sequences identified using the pin arrays are involved in the interaction of αB-crystallin with desmin filaments.

**Figure 1:**
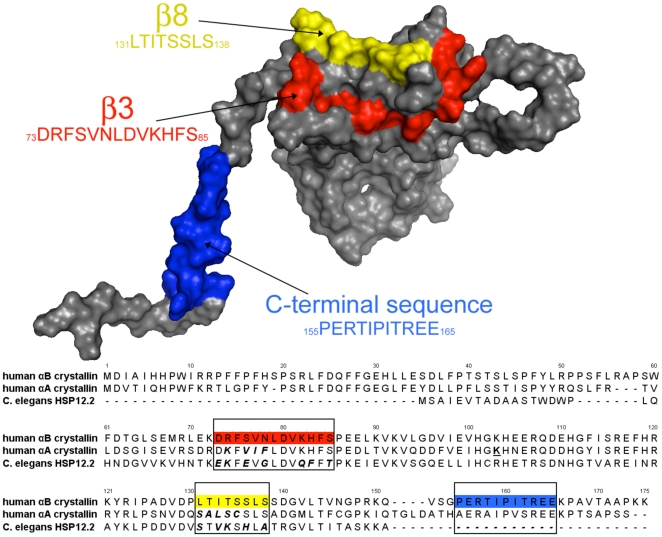
Location of IF interactive domains in β3-strand (red), β8-strand (yellow), and C-terminal 155–165 residues (blue) of human wild-type αB-crystallin. The primary sequences for human wild type αB-crystallin, human wild type αA-crystallin, and *C. elegans* wild type HSP12.2 were aligned using the residue numbers for human αB-crystallin in ClustalX. The boxes and colors in the αB-crystallin sequence correspond with the interactive sequences labeled on the surface of the 3D model. The amino acid substitutions in the αB-crystallin protein constructs are indicated by ***bold-italics***. The β3-strand of αB crystallin, _73_DRFSVNLDVKHFS_85_, was replaced with the corresponding sequences from αA-crystallin, DKFVIFLDVKHFS (αAβ3), or HSP12.2, EKFEVGLDVQFFT (CEβ3). The β8-strand of αB-crystallin, _131_LTITSSLS_138_, was replaced with the corresponding sequences in αA-crystallin, SALSCSLS (αAβ8), or HSP12.2, STVKSHLA (CEβ8). The 155–165 residues were deleted in αB-crystallin to create the Δ155–165 protein construct. The CEβ3, CEβ8, and Δ155–165 αB-crystallin protein constructs were designed to target the desmin interaction sequences.

Three αB-crystallin peptide regions (β3-strand, residues 73–85; β8-strand, residues 131–138 and the C-terminal sequences 155–165) gave some of the strongest interactions with desmin using the pin array approach [1] were selected for our studies. Recent crystallisation [24] and solution structural [25], [26] studies confirmed that all three regions are surface exposed on the αB-crystallin subunit and were potentially available to bind client proteins such as desmin (see Fig. 1). Substituting the β3- and β8-strands with the equivalent sequences from αA-crystallin and *C. elegans* HSP12.2 produced αB-crystallin protein constructs that have been well-characterised previously in terms of structural changes and client protein interactions [16], [17]. Exchange of either the β3-strand from αA-crystallin or *C. elegans* HSP12.2 with the equivalent αB-crystallin sequence was shown to have minimal effect on the secondary, tertiary and quaternary structure of αB-crystallin [17]. Replacement of the β8-strand of αB-crystallin with those from αA-crystallin and *C. elegans* HSP12.2 also did not affect secondary structure, but oligomer size was increased [16]. Likewise deleting the C-terminal sequences 155–165 altered protein oligomerisation but without significant effects upon protein secondary structure. Interestingly chaperone activity was decreased for all but one of the client proteins tested (βL-crystallin, alcohol dehydrogenase and citrate synthase) [14], [16], [17] for these five different αB-crystallin protein constructs demonstrating that all three sequences are intimately involved in and optimised for client protein recognition in αB-crystallin. Recent studies, found that both the β8-strand and C-terminal sequences, but not the β3-strand in αB-crystallin, were responsible for regulating microtubule dynamics and preventing tubulin polymerisation [2], [27]. Perhaps therefore, there are differences in the availability of the β3-strand, β8-strand and the C-terminal sequences 155–165 in αB-crystallin when a protein polymer is the client rather than individual protein subunits. For these reasons, we have determined the effect of the selected β3- and β8-strand substitutions as well as the C-terminal 155–165 deletion on the interaction of αB-crystallin with desmin filaments.

In the present study, the data demonstrate that the substituted β3-strand, β8-strand and C-terminal sequences in αB-crystallin can alter the interaction of αB-crystallin with desmin filaments and therefore all three sequences contribute to the interaction of αB-crystallin with desmin filaments. The sequence substitutions in αB-crystallin involving the *C.elegans* β3-strand and β8-strand as well as the C-terminal 155–165 deletion all caused increased desmin filament-filament interactions. In contrast, the αA-crystallin β3-strand substitution in αB-crystallin prevented desmin filament-filament interactions even more effectively than wild type αB-crystallin, but in a temperature specific manner. The data suggest that the interaction of αB-crystallin with desmin filaments does not always lead to the prevention of desmin filament aggregation, which we discuss with respect to desmin filament aggregation as a histopathological characteristic of desmin related myopathies.

## Materials and Methods

### Sequence alignment and molecular modeling

The amino acid sequence of human αB-crystallin was aligned with the sequences of human αA-crystallin and C. elegans HSP12.2 using ClustalX [28] and mapped to a 3D model (Fig. 1) for human αB-crystallin, which is in good agreement will all reported X-ray and NMR structures [18], [24], [26], [28].

### Mutagenesis of human αB-crystallin

Constructs selected for this study had either the β3 strand or β8 strand substituted by the equivalent sequences from αA-crystallin [16], [17] and C. elegans HSP12.2 [16], [17] as well as a previously characterised C-terminal deletion, which removed residues 155–165 [14]. The mutant proteins were similar in secondary structure, molecular weight and solubility to the wild-type protein. Mutagenesis of human αB-crystallin was performed using the QuikChange site-directed mutagenesis kit (Qiagen, Valencia, CA, USA) as previously described [14], [16], [17]. Two chimeric mutants in the β3 domain of αB-crystallin were created, in which the β3–strand (_73_DRFSVNLDVKHFS_85_) was swapped with the corresponding sequence from either human αA-crystallin, DKFVIFLDVKHFS (αAβ3), or *C. elegans* HSP12.2, EKFEVGLDVQFFT (CEβ3) [17]. Two chimeric mutants were created by substituting the β8-strand of human αB-crystallin, _131_LTITSSLS_138_, with the corresponding sequences from human αA-crystallin, SALSCSLS (αAβ8), or *C. elegans* HSP12.2, STVKSHLA (CEβ8) [16]. A fifth protein construct was created by deleting a C-terminal desmin interactive site (Δ155–165) [14].

### Purification of proteins

Wild type and mutant human αB-crystallin were purified from bacterial lysates as previously described using ion exchange and size-exclusion chromatography [29]. The wild type and the other five αB-crystallin protein constructs were soluble. Human desmin was purified from bacterial lysates as previously described using ion exchange chromatography [30], [31]. Wild-type αB-crystallin, the five αB-crystallin protein constructs (αAβ3, CEβ3, αAβ8, CEβ8, and Δ155–165), and desmin were purified to >97% purity as determined by SDS-PAGE.

### Assembly of desmin filaments

Assembly of desmin was performed as previously described [9], [30]. Purified Desmin at 0.2 g/l in 6 M urea, 20 mM Tris-HCl pH8, 1 mM DTT, 1 mM EDTA, 0.2 mM PMSF (in the presence or absence of αB-crystallin at 0.08 g/l) was dialysed out of urea in a stepwise fashion by reducing the urea concentration to 4 M, then 2 M, then 0 M over a period of 24 h at 22°C. Desmin assembly was then initiated by dialysis into 20 mM Tris-HCl pH7.4, 50 mM NaCl for 16 h at either 22°C, 37°C or 44°C to ensure that assembly equilibrium has been reached.

### Analysis of desmin αB-crystallin interactions by electron microscopy

Desmin, αB-crystallin and mixtures of both were diluted into assembly buffer to 100 µg/ml. A carbon film that had been coated onto freshly cleaved mica was then floated onto the surface of the sample prior to being negatively stained with 1% (w/v) uranyl acetate (Agar Scientific, UK) and retrieved with 400 mesh copper grids (Agar Scientific, UK). Grids were examined in an Hitachi H-7600 transmission electron microscope (Hitachi High-Technologies Corporation, Japan), using an accelerating voltage of 100 kV. Images were acquired using a CCD camera (Advanced microscopy Technology, Danvers, MA) and assembled into montages using Adobe® Photoshop CS (Adobe System, San Jose, CA).

Evidence of the association between desmin filaments and αB-crystallin particles in the EM images was statistically examined using likelihood ratio tests (LRT). For each combination of desmin and αB-crystallin protein construct, two representative images were selected for our analysis. Using ImageJ, a grid square was overlaid randomly over the image with a grid cell size equivalent to 11400 nm^2^. Forty cells from the 125 total were then randomly selected and the number of filaments and particles within each cell counted. We proposed that the relation between the mean number of particles in a cell, *μ*, and the number of filaments in a cell, *x*, could be well described by,
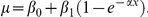



The parameter *β*_0_ describes the background density of particles, *β*_1_ is the maximum additional number of particles associated with filaments in the cell, and *α* describes how quickly each additional filament contributes particles. We also proposed that the variation in αB-crystallin particle numbers was negative-binomial distributed (NBD) to correctly account for potential variation among cells caused by unknown sources. Richards (2008) [32] provides details on how to calculate the likelihood under the assumption of a NBD. The null, which states that there is no association, is obtained by setting *α* = *β*_1_ = 0. The test-statistic is *G* = 2(LL_1_ - LL_0_), where LL_1_ and LL_0_ are the maximum logs-likelihood associated with the general model and the null model, respectively. Under the null hypothesis, *G* is chi-square distributed [33] with 2 degrees of freedom as the general model has two additional free parameters: *β*_1_ and *α*.

### Analysis of desmin, αB-crystallin interactions using centrifugation

**Figure 2:**
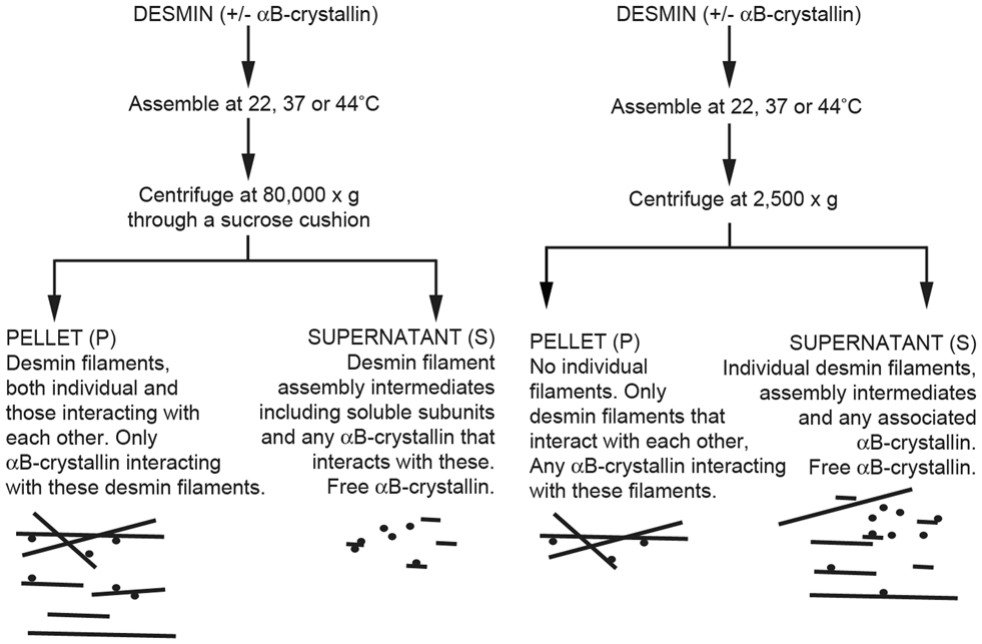
Desmin sedimentation assays. (LEFT) Schematic of the 80,000 g (high-speed) sedimentation assay [8], [9], [30]. Desmin was assembled at 22, 37, or 44°C and centrifuged at 80,000 g. The pellet (P) will contain desmin filaments and any aggregates formed as a result of filament-filament interactions. Any αB-crystallin that associates with these filaments or their aggregates will also be cosedimented. The supernatant (S) will contain soluble αB-crystallin and also any assembly intermediates or unassembled desmin. Therefore this assay measures filament assembly and αB-crystallin binding to assembled filaments. (RIGHT) Schematic of the 2,500 g (low speed) centrifugation assay. Individual desmin filaments will not be sedimented by these sedimentation conditions, neither will αB-crystallin. Only when the assembled desmin filaments self-associate into filament aggregates, will these sediment. Therefore this assay measures filament-filament interaction. If αB-crystallin binds to these aggregates, then it too will be cosedimented. Unlike the high-speed assay, it is the aggregate-associated αB-crystallin which will sediment into the pellet fraction (P) rather than the individual filaments and their associated αB-crystallin. The supernatant (S) will contain the free desmin filaments, their associated αB crystallin, desmin assembly intermediates and the unassociated αB-crystallin particles.

To investigate the interactions between desmin and the various αB-crystallin protein constructs, two separate sedimentation assays (Fig. 2) were used to separate desmin filaments and their associated αB-crystallin from un-associated αB-crystallin [9], [30]. In the high speed sedimentation assay, a 200 µl sample was layered onto a 100 µl 0.85 M sucrose cushion containing 10 mM Tris-HCl pH7.0, 50 mM NaCl, 1 mM DTT, 200 µM PMSF. Samples were then centrifuged at 30,584 rpm (RCF_max_ = 80,000×g) for 30 min at 4°C using a Beckman Coulter TLS-55 rotor (*k* factor = 50) to give pellet (Calculated size of pelletted particles ≥100 S) and supernatant fractions. The supernatant was carefully removed and samples prepared preserving volume equivalence so that a direct comparison could be made between pellet and supernatant fractions. The samples were then separated on 12% (w/v) polyacrylamide gels by SDS-PAGE and the separated proteins visualised by Coomassie Brilliant Blue staining. Destained gels were imaged using a Fujifilm LAS-100. Stained bands were quantified using Fujifilm Image Gauge V4.0 software. The protein content in the pellet (P) and supernatant (S) fractions at each temperature was measured based on Coomassie Brilliant Blue staining densities after SDS-PAGE and then plotted as bar charts to summarise the complete dataset and provide an overview. The total protein was the sum of the densities in the S and P fractions for each sample. The amount of desmin or αB-crystallin in each fraction was the density of the selected band in that fraction divided by the total protein. Low-speed centrifugation will pellet only desmin filaments that have become associated with each other by filament-filament interactions as well as any associated αB-crystallin (see (Fig. 2) and [9], [30]). This assay was initially developed to study interactions between keratin filaments [34]. Individual desmin filaments and unassociated αB-crystallin will not be pelleted under these sedimentation conditions. Immediately following assembly, samples were centrifuged at 4,900 rpm (RCF_max_ = 2,500×g) for 10 min at 20°C using an Eppendorf 5417R benchtop centrifuge and standard fixed angle rotor (F-45-30-11, *k*-factor = 377). The supernatant was carefully removed from the pellet (Calculated S-value of pelletted material ≥2258S) and as with the high-speed assay, both fractions prepared for SDS-PAGE in a way to preserve the relative protein levels in each fraction so as to allow direct comparisons to be made when viewing the stained SDS-PAGE gels. Band intensities were quantified as described above.

## Results

### Some αB-crystallin protein constructs appear to increase filament-filament interactions as seen by electron microscopy

**Figure 3:**
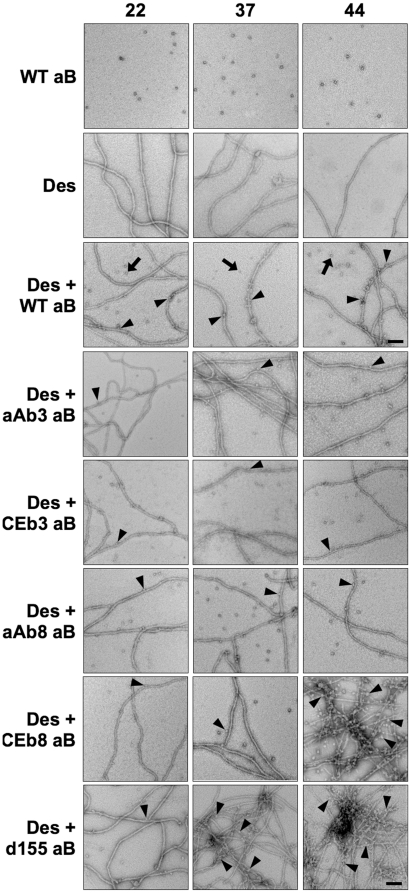
Analysis of negatively stained samples desmin and wild type αB-crystallin protein by electron microscopy. The morphology of the assembled desmin filaments and the coassembled wild type (WT aB) and various αB-crystallin protein constructs was analysed by electron microscopy. Samples were negatively stained with uranyl acetate and viewed at an 100 kV accelerating voltage. Wild-type αB-crystallin formed monodisperse particles at all three temperatures (WT aB). This was also true for all the αB-crystallin protein constructs (Fig. S1). Desmin, when assembled alone, formed long smooth 10 nm filaments at all three temperatures (Des). When desmin was assembled with wild type αB-crystallin (Des + WT aB), the filaments were not aggregated and some αB-crystallin particles were seen to associate with the filaments (arrowheads). Unassociated particles are indicated (arrows). Coassembly of desmin with either αAβ3, or CEβ3 or αAβ8 αB-crystallin gave similar results to wild type αB-crystallin at all three temperatures. In contrast, both the CEβ8 and Δ155–165 αB-crystallin protein constructs increased desmin filament-filament associations at higher temperatures leading to filament aggregation along with increased αB-crystallin particle association (arrowheads). Bar = 100 nm.

**Figure 4:**
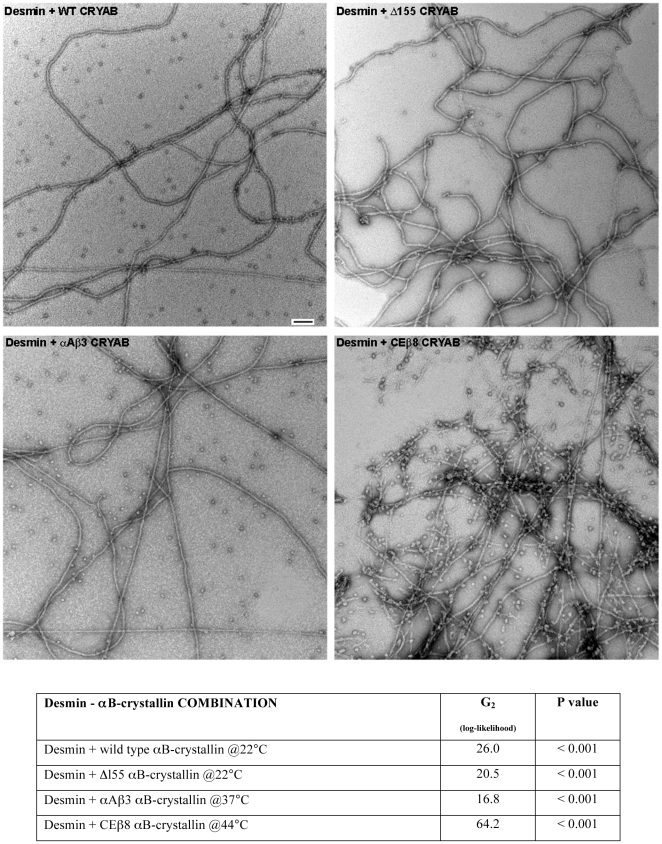
Analysis of αB-crystallin interaction with desmin filaments using a likelihood ratio test (LRT). Samples from the shown combinations of desmin and αB-crystallin were analysed by the described LRT. Examples of some of the selected images are shown. Strong statistical evidence (G2 log-likelihood scores 16.8–64.2, P values = <0.001%) was found that the αB-crystallin particles of the samples tested were positively associated with the desmin filaments (see summary table) in these samples. Bar = 100 nm.

Negative staining with uranyl acetate followed by electron microscopy was used to visualise the interactions between αB-crystallin particles and desmin filaments in samples prior to the sedimentation assays (Fig. 3). The wild type αB-crystallin formed 15–20 nm particles in agreement with observations by ourselves [8], [31] and others [35], which were seen at all three temperatures (Fig. 3; WT aB), Similarly the desmin filaments (Fig. 3; Des) had a consistent morphology at the three different temperatures typically forming 10 nm filaments many microns long. When mixed together (Fig. 3; [Des + WT aB]), both individual desmin filaments and αB-crystallin particles were readily apparent for all the various desmin-αB-crystallin combinations (Fig. 3), but now some of the αB-crystallin particles were observed to be associated with the filaments at the three different temperatures eg (Fig. 3; [Des + WT aB], arrowheads). The electron microscopic data suggest that wild type αB-crystallin particles interact with desmin filaments and this was tested for significance using LRT (Fig. 4). There was strong statistical evidence that αB-crystallin particles were positively associated with the desmin filaments (LRT; G_2_ = 26.0; *P* = ,0.001%). These data are visual confirmation that wild type αB-crystallin interacts with desmin filaments.

The various αB-crystallin protein constructs all formed particles that could be easily detected in the negatively stained samples (Fig. S1) and they did not appear to be substantially different to those formed by wild type αB-crystallin (cf Fig. 3; WT aB). When the various αB-crystallin protein constructs were included in the assembly of the desmin filaments, two observations were clear. Firstly, the presence of αB-crystallin did not appear to change noticeably the morphology of the desmin filaments (Figs. 3 and 4). As with wild type αB-crystallin, particles were clearly seen associated with desmin filaments. For the selected examples, strong evidence for the positive association of particles with desmin filaments was seen (Fig. 4). The logs-likelihood score for the interaction of the CEβ8 αB-crystallin particles with the desmin filaments at 44°C was striking (G_2_ = 64.2, *P* = <0.001%), suggesting increased interaction when compared to wild type αB-crystallin. This highlights the second of our observations. In the presence of both CEβ8 and Δ155 αB-crystallin (Fig. 3 Des + CEb8 aB and Des + d155 aB respectively) not only was there a very obvious increase in the αB-crystallin particles interacting with the desmin filaments at these higher temperatures (Fig. 3 Des + CEb8 aB and Des + d155 aB, arrowheads), but also there also appeared to be increased desmin filament-filament interactions. The effect of the various αB-crystallin protein constructs on the assembly and filament-filament interactions of desmin was then quantified by high-speed and low-speed sedimentation assays.

### Temperature dependent increase in the association of wild type αB-crystallin with desmin by low- and high-speed sedimentation assay

**Figure 5:**
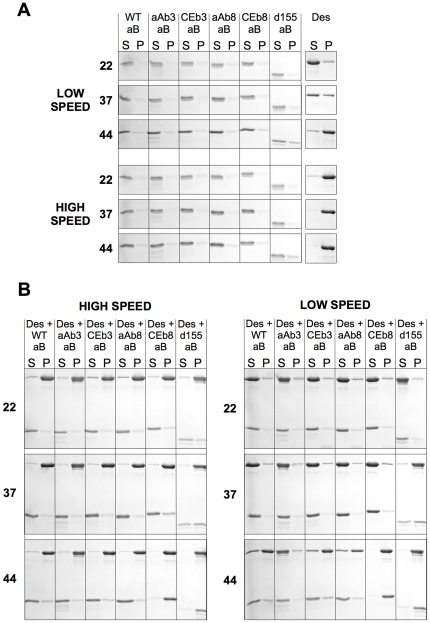
Gel electrophoretic analysis of the low- and high-speed sedimentation properties of desmin and wild type and mutant αB-crystallins. (A) The low- and high-speed sedimentation properties of each individual protein was determined at 3 different temperatures. The pellet (P) and supernatant (S) fractions were analysed by SDS-PAGE and the proportion of each protein in each fraction determined. By high-speed sedimentation assay, which measures the efficiency of desmin assembly, virtually all the desmin had pelleted at 22°, 37° and 44°C. By low-speed sedimentation assay, there was a temperature dependent increase in the proportion of desmin sedimented. The αB-crystallin remained largely in the supernatant fractions of both sedimentation assays. (B) Analysis of desmin pelleted by high- and low-speed sedimentation assay in the presence of either wild-type or the various αB-crystallin protein constructs at three different temperatures. αAβ3 αB-crystallin (aAb3 aB) reduced the proportion of desmin filaments sedimenting at low-speed at 44°C. Conversely, the CEβ8 and the Δ155–165 αB-crystallin protein constructs induced the complete low-speed sedimentation of desmin at 44°C. For each sedimentation assay, the band intensities were quantified and then combined with two other data sets to determine statistical significance and summarized in Figs. 5 and 6.

**Figure 6:**
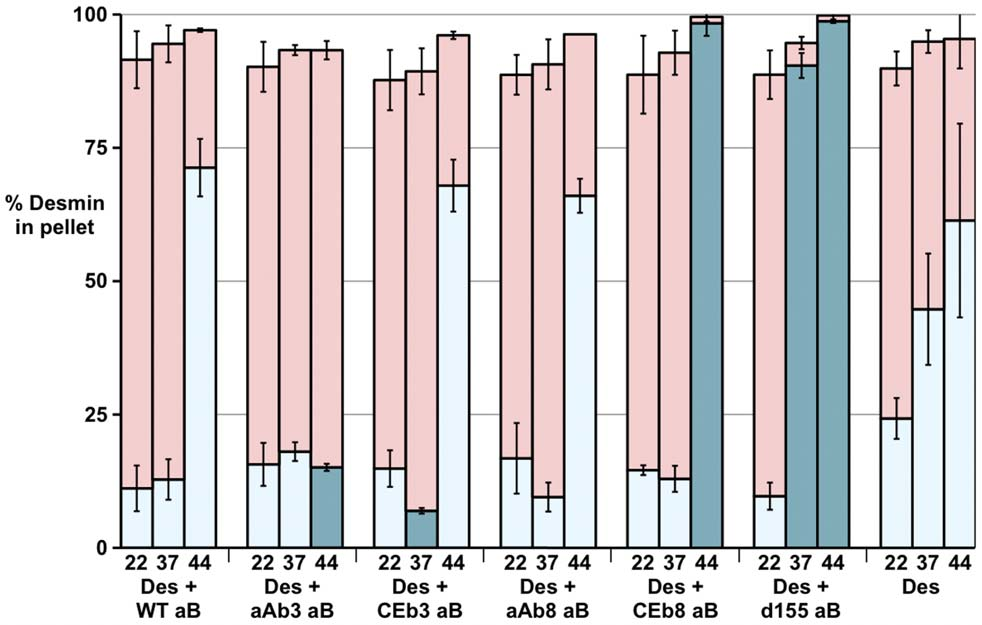
Desmin sedimentation characteristics in the presence of wild type and various αB-crystallin protein constructs. Bar chart of the low-speed (light and dark blue; lower portion of each bar) and high-speed (red) sedimentation assay data for desmin coassembled with either wild type (WT aB) or the various αB-crystallin protein constructs. The percentage of desmin in each pellet fraction at 22, 37 and 44°C was determined after both low- and high-speed sedimentation assay. The mean % from three independent experiments with its corresponding standard error was calculated for each and then plotted as a composite bar chart. The assembled desmin is pelleted by high-speed sedimentation assay. At low-speed, only the assembled filaments that have formed filament-filament interactions are pelleted. Neither temperature nor the presence of the various αB-crystallin protein constructs changed significantly the proportion of desmin pelleted in the high-speed assay. The significant differences are seen in the low-speed sedimentation assay. At 44°C, αAβ3 αB-crystallin (Des + aAb3 aB) produced a significant reduction in desmin pellet fraction (dark blue bar). Conversely, both the CEβ8 (Des + CEb8 aB) and Δ155–165 (Des + d155 aB) αB-crystallin protein constructs caused significant increases in the proportion of desmin pelleted at 44°C (dark blue bars). This was also true at 37°C for the Δ155–165 (Des + d155 aB) αB-crystallin protein construct.

Desmin filament assembly was conducted at 22°C, 37°C or 44°C in the presence or absence of the various αB-crystallin protein constructs. Pellet and supernatant fractions were analysed by SDS-PAGE and a representative experimental series is shown (Fig. 5). These data were combined with two other data sets and the % in each pellet (Figs. 5 and 6) and corresponding supernatant (data not shown) fractions calculated along with the standard error of the mean. The % of protein in the pellet fractions from both the low- and high-speed sedimentation assays from each sample are presented on the same bar in the charts (Figs. 5 and 6), with the proportion corresponding to the low speed assay being represented by the lower portion of each bar. This method of data presentation facilitates the comparison of both (high-speed and low-speed) sedimentation assays for each combination of αB-crystallin protein construct and desmin, which is needed to assess the interaction of αB-crystallin with desmin.

Desmin alone assembled efficiently and 94–98% sedimented into the pellet (P) fractions (Fig. 5A and 6). This was determined by the high-speed assay and there was no significant difference between the three different temperatures. In contrast, the low-speed centrifugation assay revealed that there was a temperature dependent increase in the proportion of desmin pelleted corresponding to 24%, 45% and 61% at 22°C, 37°C and 44°C respectively (Fig. 6, Des). This assay measured filament-filament interactions and the data therefore suggest that these interactions were temperature dependent. We excluded the possibility that desmin failed to assemble equally efficiently at the three different temperatures, because the high-speed assay revealed a similar % of desmin in the pellet fractions at the three different temperatures (Fig. 6, Des).

In the presence of αB-crystallin, 93–100% desmin sedimented into the high-speed pellet at the three temperatures indicating that wild type αB-crystallin did not alter the extent of desmin assembly (Fig. 5, HIGH SPEED; cf Des and Des + WT aB. Fig. 6; cf Des and Des + WT aB). In contrast, the low-speed sedimentation assay showed that the presence of αB-crystallin prevented filament-filament associations and only 12% of the desmin filaments sedimented at 22°C and 37°C (Fig. 5, 6. LOW SPEED; cf Des and Des + WT aB). At 44°C, the preventative effect was lost and there was no statistically significant difference in the proportion of pelletable desmin in the presence or absence of wild type αB-crystallin (Fig. 6; cf Des and Des + WT aB).

**Figure 7:**
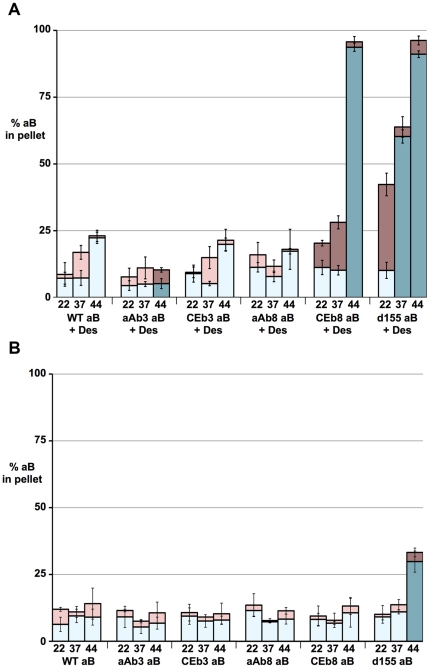
Cosedimentation of αB-crystallin with and without desmin filaments. A. **Cosedimentation of αB-crystallin with desmin filaments.** Summary of the low-speed (light blue and dark blue) and high-speed (light red and dark red) sedimentation data for various αB-crystallin protein constructs coassembled with desmin, as quantified by gel densitometry. The percentage of αB-crystallin in the pellet fractions at 22°, 37° and 44°C was determined after both low- and high-speed sedimentation to quantify the association of αB-crystallin with the sedimented desmin filaments. The most striking observation is that both the CEβ8 (CEb8 aB + Des) and Δ155–165 (d155 aB + Des) αB-crystallin protein constructs showed significant increases in desmin binding at 44°C as shown by the high speed assay (44°C, dark red bars). Conversely, αAβ3 αB-crystallin (aAb3 aB + Des) showed significantly decreased association at 44°C at high speed. The CEβ3 (CEb3 aB) and αAβ8 (aAb8 aB) protein constructs showed similar sedimentation properties to wild type αB-crystallin. B. **Aggregation of wild type and mutant αB-crystallins as measured by low- and high-speed sedimentation.** Summary of the low-speed (blue) and high-speed (red) sedimentation data for the various αB-crystallin protein constructs as quantified by gel densitometry. The percentage of αB-crystallin in the pellet fractions at 22°, 37° and 44°C was determined after both low- and high-speed sedimentation assay to quantify the aggregation of the αB-crystallins. All the other protein constructs showed similar sedimentation properties to the wild type (WT aB) αB-crystallin, except Δ155–165 (d155 aB) αB-crystallin at 44°C, which showed increased aggregation by both low- (darker blue) and high-speed (darker red) sedimentation assay.

These experiments also measured the proportion of αB-crystallin that co-sedimented with the desmin filaments (Fig. 7A). In the absence of desmin, wild type αB-crystallin remains almost entirely in the supernatant fractions of both the high- and low-speed centrifugation assays (Figs. 4 and 6B). In the presence of desmin, however, there is a temperature dependent increase in the proportion of wild type αB-crystallin in the pellet fractions from the high-speed sedimentation assay (Figs. 4 and 6A). This corresponded to 9%, 17%, and 23% of the wild type αB-crystallin at 22°C, 37°C and 44°C respectively. These data confirm observations made in a previous study [7].

These data form the baseline for assessing the effects of changing the β3-strand, β8-strand sequences and deleting residues 155–165 in wild type αB-crystallin on the association of αB-crystallin with the desmin filaments and the subsequent effects on filament-filament interactions. All the various αB-crystallin protein constructs were soluble as determined by both sedimentation assays (Fig. 7B) and formed mono-disperse particles as judged by electron microscopy (Fig. S1). Only the Δ155–165 αB-crystallin protein construct showed an increased tendency to pellet and then only at 44°C (Fig. 7B, d155 aB).

### Substituting the β8 strand of αB-crystallin and deleting the C-terminal residues 155–165 can promote desmin filament-filament interactions rather than inhibiting them

By comparison to the β3-strand substitutions, changing the β8-strand produced αB-crystallin protein constructs that showed either no significant improvement (αA-crystallin β8 chimera αB-crystallin (aAb8) see Figs. 4, 5 and 6A) or a very obvious increase in desmin filament-filament interactions (*C. elegans* HSP12.2 β8 chimeraαB-crystallin (CEb8)), the most dramatic observed at 44°C (Fig. 5B and 6, LOW SPEED; [Des + CEb8 aB]). In fact reduction in the ability of this *C. elegans* HSP12.2 β8 chimera αB-crystallin to prevent desmin filament-filament interactions was mirrored by the deletion of the C-terminal residues 155–165 (Figs. 4B and 5, LOW SPEED; [Des + d155 aB]). It was also apparent from the high-speed centrifugation assay that a very striking shift in the pelletable portion of both these αB-crystallin constructs when in the presence of desmin filaments (Figs. 4B and 6A. HIGH SPEED; [Des + CEb8 aB] and [Des + d155 aB]). Quantification of the proportion of αB-crystallin in the pellet fractions (Fig. 7A; [Des + CEb8 aB] and [Des + d155 aB]) demonstrated that in excess of 95% of these αB-crystallin proteins had indeed co-sedimented with the desmin filaments. For the Δ155–165 protein construct this might be partly due to the increased aggregation of this αB-crystallin at 44°C, but this is not the case for the 37°C sample, a temperature at which this protein construct did not obviously aggregate (Fig. 7B; d155 aB). For the CEb8 protein construct, no temperature dependent self-aggregation was observed (Fig. 7B; CEb8 aB) and therefore the increased cosedimentation of the CEβ8 construct with the desmin filaments seen by low- and high-speed centrifugation assay is due to an increased association of this αB-crystallin with the desmin filaments at 44°C (Figs. 4B and 6A; [Des + CEb8 aB]). The consequence of these increased interactions for both the CEβ8 and Δ155–165 αB-crystallin constructs with the desmin filaments manifested itself as very obvious increased degree of desmin filament-filament associations (Fig. 6, blue bars for [Des + CEb8 aB] and [Des + d155 aB] compared with [Des + WT aB]) and also seen by electron microscopy (Fig. 3; [Des + CEb8 aB] and [Des + d155 aB]). The aggregation of the desmin filaments observed with these two αB-crystallin constructs at 44°C is very apparent when compared to the other representative images in Fig. 3. We conclude from these data that the β8 region and the C-terminal 155–165 residues also play an important role in the interaction of αB-crystallin with desmin filaments. The β8-strand substitution with the *C. elegans* HSP12.2 sequences and the deletion of the C-terminal residues 155–165 in αB-crystallin have both caused an increase in the interaction of αB-crystallin with the desmin filaments.

### Substitution of the β3 strand can increase the ability of αB-crystallin to inhibit desmin filament-filament interactions

In contrast to the *C. elegans* HSP12.2 β8-strand substitution and deletion of the C-terminal 155–165 residues, substituting the β3–strand in the wild type αB-crystallin with that from αA-crystallin caused a dramatic decrease in the proportion of sedimentable desmin at 44°C in the low-speed assay (Fig. 6 [Des + aAb3 aB] dark blue bar cf [Des + WT aB], light blue bar). The effect was very evident by the gel analysis of the 44°C samples (Fig. 5B, LOW SPEED cf Des + aAb3 aB and Des + WT aB). Quantification of these gel data (Fig. 6, Des + aAb3 aB) showed that there was a 5 fold difference in the sedimentable desmin by low-speed sedimentation assay (Fig. 6 [Des + aAb3 aB] dark blue bar cf [Des + WT aB], light blue bar). The results indicated a significant reduction in the extent of the filament-filament interactions in the presence of the αAβ3-αB-crystallin protein construct. The high-speed sedimentation assays showed no change in the level of assembled desmin at 44°C when coassembled with the αAβ3 αB-crystallin protein construct (Fig. 5B and 6. HIGH SPEED, Des + aAb3 aB) compared to desmin assembled in the presence of wild type αB-crystallin (Fig. 5B and 6. HIGH SPEED, Des + WT aB) in support of this conclusion.

Although the effect of substituting the β3-strand from *C.elegans* HSP12.2 (CEβ3) was not as dramatic compared to that with the equivalent sequences from αA-crystallin (αAβ3), there was still a significant reduction (∼2 fold) in the level of desmin sedimented in the low-speed centrifugation assay at 37°C (Fig. 5B and 6; LOW SPEED [Des + CEb3 aB] cf LOW SPEED [Des + WT aB]). At both 22°C and 44°C, no additional effects for the CEβ3 strand substitution into αB-crystallin were apparent (Figs. 5B and 6, LOW SPEED; cf [Des + CEb3 aB] and [Des +WT aB]). Once again this was not because of any effects of the αB-crystallin protein constructs upon desmin assembly *per se* as there was no significant difference in the pelletable desmin by high-speed sedimentation assays (Figs. 5B and 6 HIGH SPEED). Electron microscopy confirmed that the morphology of the desmin filaments and the αB-crystallin particles were similar to those seen in samples containing the wild type αB-crystallin and desmin (Fig. 3 cf [Des +WT] with {Des + CEb3 aB]).

The combined data show that substituting the β3-strand of αB-crystallin can have beneficial effects for the ability of αB-crystallin to prevent desmin filament-filament interactions. The effects are temperature specific, but these data demonstrate that it is possible to improve this activity by changing the β3-strand, confirming that this domain plays an important role in the association of αB-crystallin with desmin filaments.

### The role of αB-crystallin binding in the prevention of desmin filament-filament interactions

The proportion of αAβ3 that cosedimented with desmin filaments in the high-speed centrifugation assay at 44°C was significantly lower than that seen for the wild type αB-crystallin (Fig. 7A, 44°C [aAb3 aB + Des] and [WT aB + Des]). We interpret this to indicate reduced binding to the filaments. This was in addition to the ability of αAβ3 to prevent desmin filament-filament associations at 44°C, which was increased some 5 fold compared to wild type (Fig. 6, 44°C [Des + aAb3 aB] and [Des + WT aB]). Conversely, the proportion of CEβ8 that cosedimented with desmin filaments in the high-speed centrifugation assay at 44°C was significantly higher than that seen for the wild type αB-crystallin (Fig. 7A, 44°C [CEb8 aB + Des] and [WT aB + Des]), indicating increased binding to the filaments. The ability, however, of CEβ8 αB-crystallin to prevent desmin filament-filament interactions at 44°C was decreased compared to wild type αB-crystallin (Fig. 6, 44°C blue bars [Des + Ceb8 aB] and [Des + WT aB]). Therefore the ability to bind to desmin filaments does not necessarily correlate with the prevention of desmin filament-filament interactions. More specifically, increased αB-crystallin binding was not necessarily correlated with increased effectiveness in the prevention of filament-filament interactions.

## Discussion

### Implications for αB-crystallin interactions with desmin filament

**Figure 8:**
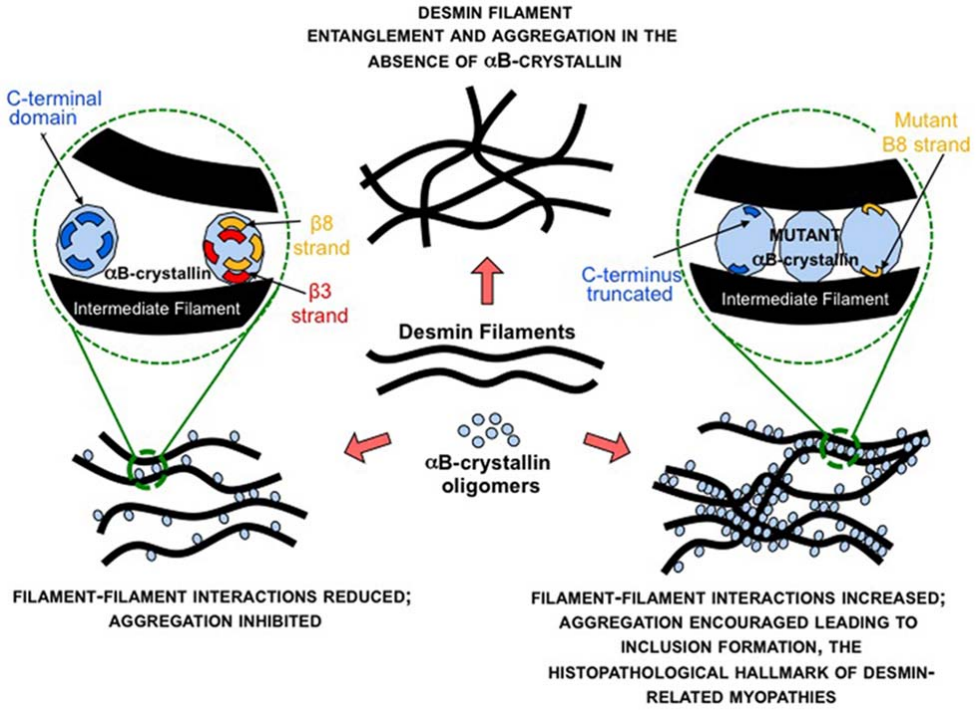
Summary of the influences of αB-crystallin on desmin filaments. The β3- and β8-strands and the Δ155–165 sequences (C-terminal domain) in αB-crystallin were identified from peptide array studies as being desmin interaction sequences. In wild type αB-crystallin these sequences contribute to the interaction of the αB-crystallin oligomers with desmin filaments to prevent their self-association and the formation of filament-filament aggregates. This activity can be increased by substituting the β3-strand from other small heat shock proteins (αA-crystallin and *C. elegans* HSP12.2). Substituting the β8-strand in αB-crystallin or removing the 155–165 residues appears to lead to the loss of this activity, but increases the binding of αB-crystallin to desmin filaments. This in turn will encourage increased filament-filament interactions, which in the case of the many point mutations in αB-crystallin linked to inherited myopathies, then leads to protein inclusion formation and the appearance of the histopathological feature of desmin-related myopathies – protein inclusions containing both desmin and αB-crystallin.

The data presented here are the first analysis of the regions in αB-crystallin previously identified as capable of interacting with desmin filaments. The strand-swapping approach used here has confirmed that the β3-strand is an important interaction site in αB-crystallin for desmin filaments. A 5 fold improvement at 44°C in preventing filament-filament interactions for the aAb3 protein construct is a direct measure that this sequence is indeed important. Equally dramatic results were also obtained for one of the β8 strand-swapped protein constructs (CEb8) and for the C-terminal deletion of residues 155–165. For these two protein constructs (CEb8 and d155), a significant decrease in the ability of αB-crystallin to prevent desmin filament-filament interactions was observed (Fig. 6). The results emphasise the importance of all three regions to the interactions between αB-crystallin and self-assembling desmin filaments and subsequent filament-filament interactions (Fig. 8).

We also conclude from our data that it is reasonable to expect that changes to any interacting region can potentially produce positive as well as negative effects upon the observed activities of αB-crystallin toward desmin filaments (Fig. 8). Indeed summarizing the activity of the β3-strand, β8-strand and Δ155–165 αB-crystallin protein constructs toward other client proteins shows that both improvement and deterioration should be expected with such changes to client protein binding sequences (Table 1). Currently the data presented here do not distinguish between a direct or indirect interaction of these regions with the desmin filaments. Nevertheless the fact that αB-crystallin particles are seen to decorate desmin filaments (Fig. 3) and the various αB-crystallin protein constructs cosedimented to either greater or lesser extents (Figs. 4 and 5), we interpret to mean that αB-crystallin binds directly to the desmin filament and involves the β3-strand, β8-strand and Δ155–165 sequences of αB-crystallin

**Table 1:** Comparison of the chaperone activities of WT and the various αB-crystallin protein constructs used in this study with different client proteins.

	insulin (DTT-induced)			β crystallin	ADH	CS	median # of subunits	Far-UVCD		Near-UVCD	
	22°C	37°C	44°C	50°C	50°C	50°C	22°C	37°C	50°C	37°C	50°C
**no αB**	1.00	1.00	1.00	1.00	1.00	1.00	x	x	x	x	x
**WT**	0.85	0.91	0.94	0.16	0.13	0.59	24	-	-	-	-
**αAβ3**	0.91	0.73	0.77	0.25	0.22	0.51	27	UC	UC	UC	UC
**CEβ3**	0.95	1.03	1.09	0.49	0.42	0.69	27	UC	UC	UC	UC
**αAβ8**	0.95	0.86	0.95	0.43	0.74	0.61	31	UC	UC	UC	UC
**CEβ8**	0.93	0.87	0.87	6.09	0.99	0.62	31	UC	UC	UC	UC
**Δ155**	1.02	133	1.28	11.33	2.75	0.81	25	UC	UC	UC	UC

Numbers shown are normalised values of protein aggregation as determined by light scattering. Aggregation was calculated as (light scattering in the presence of chaperone)/(light scattering in the absence of chaperone) for the relevant client proteins. Where normalised values became greater than 1.00, this indicates increased protein aggregation. The results summarized here for the various αB-crystallin protein constructs are from previously published work (αAβ3 and CEβ3 see reference [17]; αAβ8 and CEβ8 see reference [16]; Δ155 see reference [14]). Median number of subunits was determined by size exclusion chromatography. Data from far- and near-UVCD spectroscopy were used to analyze secondary and tertiary structure of the αB-crystallin protein constructs. All αB-crystallin proteins consisted of large polydisperse oligomers and had far- and near-UVCD spectra unchanged (UC) from wild type (WT) αB-crystallin at 37°C and 50°C.

These results therefore confirmed the pin array studies that found multiple sites were responsible for the interaction of αB-crystallin with desmin [1]. Indeed the analysis of the effects of the cardiomyopathy causing mutant R120G αB-crystallin adds to this argument as this residue is outside of the β8-strand studied here, but is part of the β7-strand identified from the pin-arrays to be involved also in binding to desmin [1]. In patients, the R120G mutation leads to desmin filament aggregation and the formation of characteristic inclusions that were also enriched in αB-crystallin [5]. It was subsequently shown that the mutation also increased the binding affinity of αB-crystallin to desmin filaments by increasing the Kd by some two fold [7].

### Relevance of desmin filament binding to histopathological aggregates of desmin and αB-crystallin

Another feature to emerge from the data presented here is that a significant increase in the binding of αB-crystallin to desmin filaments does not necessarily result in a similar increase in the ability of αB-crystallin to prevent the self-association of desmin filaments. This observation was first made with R120G αB-crystallin and GFAP filaments using a simplified viscometry assay [36] and was later confirmed for desmin filaments using the low-speed sedimentation assay [7]. Both the CEβ8 and Δ155–165 αB-crystallin protein constructs studied here showed increased co-sedimentation with desmin filaments by high-speed sedimentation assay (Fig. 7A). Electron microscopy (Fig. 3) revealed this was direct binding to the desmin filaments, which coincided with an increase in desmin filament-filament associations as measured by the low-speed centrifugation assay (Fig. 6). In contrast, the αAβ3 αB-crystallin construct significantly inhibited desmin filament-filament associations, but the cosedimentation of this protein construct with desmin filaments was also significantly decreased at 44°C (Figs. 4–5). Compare these data to the detailed analysis of the Q151X myopathy-causing mutation in αB-crystallin where increased desmin cosedimentation of Q151X αB-crystallin was accompanied by a very significant decrease in desmin filament-filament associations [31]. The current study therefore confirms the importance of the β3- and β8-strands in interactions with desmin filaments. It also adds to previous observations that increased binding of αB-crystallin to desmin filaments does not necessarily correlate with the prevention of desmin filament-filament associations. This is reminiscent of the situation in desmin related myopathies where the characteristic histopathological feature of the disease is protein aggregates containing both αB-crystallin and desmin [11], [12], [13].

### A role of αB-crystallin in modulating interactions between biopolymers?

Further quantitative studies are required to define the relationship(s) between binding of αB-crystallin and the polymerisation and subcellular distribution of important biopolymers such as intermediate filaments, microtubules and actin filaments. This poses an obvious question concerning the selection or hierarchy in the interaction of αB-crystallin with the different polymers and how this involves the different interaction sequences.

For the client protein T4 lysozyme, αB-crystallin has both high and low affinity binding sites [37], [38]. Binding to the high affinity site appeared to induce structural changes in the client protein itself. For desmin, the measured dissociation constant [7] is equivalent to the low affinity site on T4 lysozyme, but how this might affect the subunit geometry within the filament has not yet been determined. The pin array studies show that the β3-strand, β8-strand and 155–165 regions are all involved in binding to all three cytoskeletal proteins [1]. Refinement of these studies has shown that peptides derived from the β8-strand and the C-terminal 15–165 region could inhibit tubulin assembly whereas the sequences in the β7–strand and including R120 actually promoted tubulin assembly [2]. Interestingly the β3-strand was not involved in the binding to microtubules, but the studies here have identified this region as a desmin filament interacting domain. This offers the possibility that the combination of interaction sites could be a key to polymer recognition. The role of αB-crystallin in the self-assembly of biopolymers and particularly the three main cytoskeletal elements [39], remains to be fully determined.

## Supporting Information

Figure S1 Electron microscopy characterisation of the αB-crystallin proteins used in this study. The αB-crystallin samples were stained with 1% (w/v) uranyl acetate and then processed for electron microscopy. All proteins appeared as monodisperse particles. Scale bar represents 100 nm.
(TIF)
