# Supplementary figures and images for: Multiple Sites in αB-Crystallin Modulate Its Interactions with Desmin Filaments Assembled In Vitro
Source: PLoS One. 2011 Nov 9;6(11):e25859. doi: 10.1371/journal.pone.0025859 (PMC3212511; doi:10.1371/journal.pone.0025859)

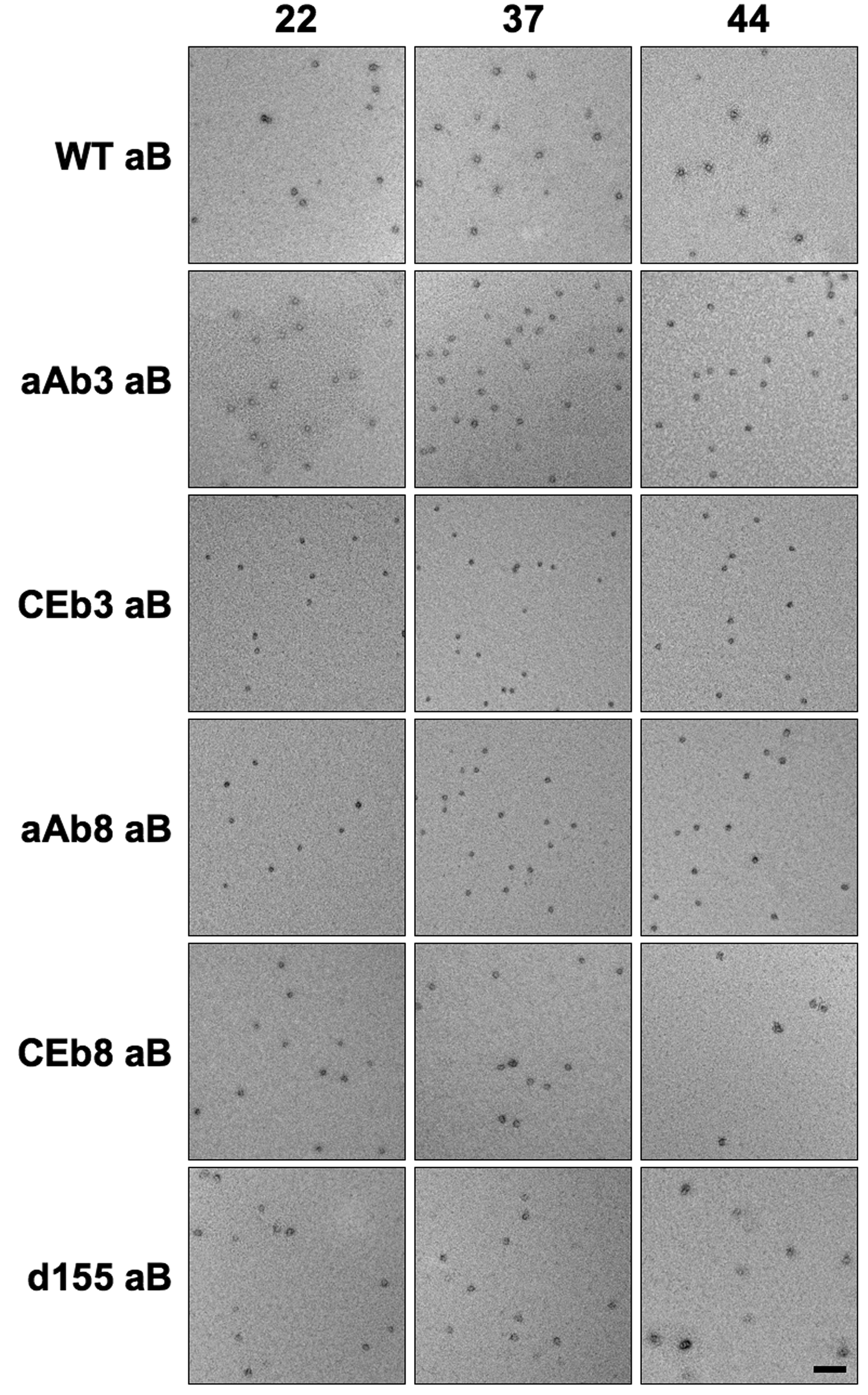

Supplement: Figure S1 — Electron microscopy characterisation of the αB-crystallin proteins used in this study. The αB-crystallin samples were stained with 1% (w/v) uranyl acetate and then processed for electron microscopy. All proteins appeared as monodisperse particles. Scale bar represents 100 nm. (TIF) [file pone.0025859.s001.tif]
